# Hippocampal Calcification on Computed Tomography in Relation to Cognitive Decline in Memory Clinic Patients: A Case-Control Study

**DOI:** 10.1371/journal.pone.0167444

**Published:** 2016-11-28

**Authors:** Remko Kockelkoren, Jill B. De Vis, Willem P. Th. M. Mali, Jeroen Hendrikse, Pim A. de Jong, Annemieke M. Rozemuller, Huiberdina L. Koek

**Affiliations:** 1 Department of Radiology, University Medical Center Utrecht, Utrecht, The Netherlands; 2 Department of Pathology, University Medical Center Utrecht, Utrecht, The Netherlands; 3 Department of Geriatrics, University Medical Center Utrecht, Utrecht, The Netherlands; McGill University, CANADA

## Abstract

**Background:**

It was recently shown that calcification of the hippocampus can be detected on computed tomography (CT) images and these calcifications occur in up to 20% of people over 50 years of age. However, little is known about hippocampal calcification and its relation to cognition and cognitive decline. Therefore, the aim of this study was to (1) determine the prevalence of hippocampal calcification on CT in memory clinic patients controls, and (2) to assess its relation with cognitive decline.

**Methods:**

67 patients from a memory clinic (cases) were matched by age and gender to a control group. In both groups, hippocampal calcification was assessed by two raters on thin slice, non-contrast enhanced brain CT images. Calcifications were scored bilaterally on presence and severity (absent, mild, moderate, severe). Mini Mental State Exam (MMSE) score was determined in cases.

**Results:**

Hippocampal calcification presence was significantly higher in cases (N = 26, 38.8%) compared to controls (N = 9, 13.4%) (P < .01) with an odds ratio of 4.40 (95%CI: 1.63–14.87). In cases, MMSE score was significantly lower in those with hippocampal calcification compared to those without (21.6 vs 24.5, p = .02).

**Conclusion:**

In this case-control study we found significantly more hippocampal calcification in patients with cognitive decline as compared to controls. Furthermore, within the cases, MMSE score was significantly lower in those with hippocampal calcification.

## Introduction

Calcification of the hippocampus as seen on computed tomography (CT) is a relatively unknown radiological finding, frequently overlooked or misinterpreted as calcification of the adjacent choroid plexus.[1] Due to the increased image quality of CT scanners and more common use of thin slice imaging and multiplanar reformatting techniques, choroid plexus calcification and hippocampal calcification (HCC) can now be viewed independently. Applying these improved CT techniques, the first radiological study on this subject showed that HCC is a relatively frequent finding in the elderly occurring in up to 20% of subjects over 50 years of age.[1]

HCC has previously been described in histopathological literature where it was postulated that HCC is related to Vascular Fibrosis and Calcification (VFC) of the hippocampus, a form of vascular pathology which results in neural loss in affected subjects.[2] Despite this finding no further studies investigated HCC and its possible relation to cognition and cognitive decline. One important factor which could contribute to this is the fact that magnetic resonance imaging (MRI), the main imaging modality in neurocognitive research, is considerably limited in visualizing calcifications.[3]

The new insights gained in the detection of HCC with CT, the high prevalence of HCC, and the knowledge of VFC being related to neural loss have led us to investigate the relation between HCC and cognitive decline. In this pilot case-control study the prevalence of HCC in patients referred to a memory clinic for cognitive problems was compared with the prevalence of HCC in control subjects, and the relation of HCC with cognitive function was studied.

## Methods

### Patients

Our institutional review board (Medical Ethical Testing Committee) approved this study (approval number: 15/432). Informed consent was waived due to the retrospective nature of the study. Inclusion criteria for the cases were; (1) patients referred to our memory clinic between 2009 and 2015 because of cognitive complaints and (2) a non-contrast enhanced brain CT examination within one year of the visit to the memory clinic. In the subjects referred to the memory clinic, global cognitive function was assessed by the treating physician using the Mini Mental State Examination (MMSE).[4] Furthermore, as it is known that MMSE scores can be influenced by education level we obtained information on the number of years of education completed.

The control group consisted of high energy trauma (HET) patients who were examined in our center between 2011 and 2015. Trauma patients were chosen over other patient groups as they represent a random sample of the general population due to the random nature of traumas. In our center all HET patients undergo a total body CT scan to exclude intracranial pathology. Patients with extensive intracranial hemorrhage on CT were excluded from the study. Hospital medical records of all control patients were searched for history of dementia and other possible causes for cognitive decline. The controls were matched by gender and age to the cases with a maximum age-difference at the time of the CT of one year.

### CT examinations

**Fig 1:**
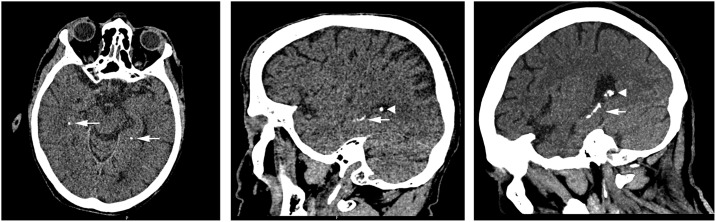
Hippocampal calcification on thin unenhanced CT examinations (arrows). The left axial reconstructed image shows bilateral mild hippocampal calcification (dot). The middle sagittal reconstructed image shows moderate hippocampal calcification (multiple dots). The right sagittal reconstructed image shows severe hippocampal calcification (confluent). Calcification of the choroid plexus is also visible (arrowheads).

Brain CT examinations were acquired on a Philips Brilliance 64-slice or 256-slice CT scanner (Philips Healthcare, Best, The Netherlands). Patients were scanned from the skull base to the vertex. The non-contrast enhanced thin slice reconstructions (0.625–1 mm) were rated blinded and individually by two experienced radiologists with 14 and 16 years of experience in reading (brain) CT scans. Images were analyzed in axial, coronal and sagittal plane in the brain window setting (Center: 40, Width: 100) using the Philips IntelliSpace Portal 7.0 (Philips Healthcare, Best, The Netherlands). Calcifications were scored bilaterally in the hippocampus as absent, mild (one dot), moderate (multiple dots) or severe (confluent) (Fig 1). Calcifications are seen in the brain CT window setting as white (clustered) dense configurations comparable to bone and had to be clearly located in the hippocampus. In case of disagreement between both readers, a consensus reading determined the final score.

### Statistical analyses

Descriptive statistics, means/medians and 95% confidence intervals for continuous variables and counts and percentages for categorical variables were applied to describe the characteristics of the cohort. Spearman’s rank correlation coefficient was used for correlation analyses. McNemar’s test for paired nominal data was used to compare HCC presence in cases and controls and Students t-tests were used for determining differences in continuous variables. Chi-square test was used to determine the difference in education level. Interobserver agreement of HCC presence (yes/no) was calculated using kappa statistics.[5] A p-value of <0.05 was considered significant. Statistical analysis was performed using SPSS (IBM SPSS Statistics, Version 23.0. IBM Corp, Armonk, NY).

## Results

The final cohort consisted of 67 cases, 32 men and 35 women and an equal number in the control group. The median number of days between the memory clinic visit and brain CT examination was 60 (range 0 to 329 days). Cases and controls were 77.2 (range 49–95 years) and 76.8 years of age (range 49–95 years) respectively. The mean age of women (79.1 years) was significantly higher than that of men (74.7 years) (P = .033). Most frequent diagnoses of memory clinic patients was Alzheimer’s disease (n = 24 [35,8%]), vascular dementia (n = 8 [11,9%]), combined Alzheimer’s/vascular dementia (n = 11 [16,4%]) and mild cognitive impairment (n = 9 [13,4%]) (Table 1). Four control patients had a previous diagnosis of possible cognitive decline. Namely, early dementia, vascular dementia, possible dementia and Pick’s disease.

**Table 1:** Baseline characteristics memory clinic patients and controls.

	Memory Clinic Group (n = 67)	Control Group (n = 67)
**Age (mean, range)**	77.2 (49–95)	76.8 (49–95)
**Gender (female)**	35 (52)	35 (52)
**Primary Diagnosis**		
Alzheimer’s disease	24 (36)	0 (0)
Vascular dementia	8 (12)	1 (25)
Combined*	11 (16)	0 (0)
MCI	8 (12)	0 (0)
SCI	2 (3)	0 (0)
Other dementia	6 (9)	1 (25)
Dementia NOS	2 (3)	2 (50)
Other diagnosis	6 (9)	0 (0)

*Combined Alzheimer’s disease and vascular dementia.

MCI = Mild cognitive impairment, SCI = Subjective cognitive impairment.

NOS = Not otherwise specified. Values are presented as n (%)

HCC was observed more often in cases (N = 26 [38.8%]) compared to controls (N = 9, [13.4%]), with an odds ratio of 4.40 (95%CI: 1.63–14.87, p<0.01) (Table 2). In the cases the distribution of severity of HCC was 13 mild, 6 moderate and 7 severe and in the controls 3, 1 and 5, respectively (Table 3). Mild calcifications were always found in the hippocampal tail while moderate and severe calcifications extended further towards the hippocampal head. The calcifications were located in the lateral segment of the hippocampus adjacent to the temporal horn of the lateral ventricles. There was no significant difference in the prevalence of HCC between men and women in the cases and controls being 46,9% and 31,4% (p = .195) and 9.3% and 17.1% (p = .352), respectively.

**Table 2:** Hippocampal calcifications in cases and controls by age and gender.

	Memory Clinic Group		Control Group	
	Patients	HCC	Patients	HCC
**Age (years)**				
<70	14 (21)	2 (14)	14 (21)	3 (21)
70–80	26 (39)	12 (46)	26 (39)	1 (4)
>80	27 (40)	12 (44)	27 (40)	5 (19)
Total	67 (100)	26 (39)	67 (100)	9 (13)
**Gender**				
Male	32 (48)	15 (47)	32 (48)	3 (9)
Female	35 (52)	11 (31)	35 (52)	6 (17)

HCC = Hippocampal Calcification. Values are presented as n (%)

**Table 3:** Hippocampal calcifications severity and laterality in cases and controls.

	Memory Clinic Group	Control Group
**HCC Severity**		
Absent	41 (61)	58 (87)
Mild	13 (19)	4 (6)
Moderate	6 (9)	3 (4)
Severe	7 (10)	2 (3)
**HCC Side**		
Left	2 (8)	1 (11)
Right	9 (35)	3 (33)
Bilateral	15 (57)	5 (56)

HCC = Hippocampal Calcification. Values are presented as n (%)

In cases and controls combined, HCC severity increased with age (r = .20, P = .02). Patients with HCC were on average older (79.6 years) as compared to patients without HCC (76.1 years) (P = .04). Calcifications occurred most frequently bilaterally (N = 20 [57.1%]), followed by the right hippocampus (N = 12 [34.3%]) and left (N = 3 [8.6%]).

MMSE was available in 56 cases and was lower in patients with HCC (21.6 points [95%CI 19.3–23.9]) compared to those without (24.5 points [95%CI 23.1–25.9], p = .02). There was no significant difference in completed years of education between these 56 memory clinic patients with and without HCC (P = .66) (Table 4).

**Table 4:** Completed years of education in memory clinic patients with MMSE.

Education in years	HCC	No HCC
0–4	0 (0)	0 (0)
5–7	4 (20)	10 (28)
8–12	7 (35)	14 (39)
13–17	9 (45)	12 (33)

HCC = Hippocampal Calcification, MMSE = Mini Mental State Examination. Values are presented as n (%)

Interobserver agreement of HCC presence was good with a kappa of .80.

## Discussion

In this pilot case-control study we showed that HCC as seen on CT images is three times more common in patients visiting a memory clinic for cognitive problems compared to control patients, and that cognitive functioning in these patients, as measured by MMSE, is worse when HCC is present.

Calcification of the hippocampus is still relatively unknown and has only been examined in two studies, one from 2002 focusing on histopathology[2] and one from 2012 on CT imaging.[1] In the histopathological study it was shown that hippocampal VFC occurred in up to 60% of patients with Alzheimer’s disease and 40% of a control group. In this study it was found that VFC always started in the tail of the hippocampus and only progressed in a limited number of cases to the body and head. The calcifications were located in the wall of precapillary and capillary vessels within the flow territory of the middle hippocampal artery and were described as ‘idiopathic non-atheriosclerotic’. In the more severe cases VFC led to loss of vessel wall structure and disappearance of nearly all neurons in the dentate gyrus, CA1 sector and subiculum proper.[2] The authors concluded that VFC in the hippocampus may contribute to or cause hippocampal sclerosis, a disease that has been linked to cerebrovascular pathology and dementia in the elderly.[6,7]

Thus far only one CT imaging study looked into HCC and concluded that it is a common finding in patients older than 50 years, contradicting previous radiological literature which suggested that HCC only occurred in rare conditions.[1] They further concluded that thinslice, multiplanar CT allows for adequate distinction between choroid plexus calcification and HCC, which was confirmed by the good interrater agreement in our study (kappa: .80).[1] Unfortunately, within their study, analysis of the effect of calcification on cognitive functioning was hindered due to small sample size and absence of cognitive testing. Interestingly, calcifications as visualized by CT were also found to always start in the tail of the hippocampus and in some cases progress to the body and head. Thereby, it was hypothesized that the HCC seen on CT scan could be caused by VFC. In our study HCC, and VFC also appeared to be located in the same segment within the hippocampus. HCC was found throughout the lateral segment of the hippocampus which correlates to the dentate gyrus and CA1 sector, the area where the calcified vasculature was described by Wegiel et al in their histological study.[2] If HCC and VFC, two calcifying processes that originate in the hippocampal tail, were to be related it could also explain the apparent relation we found in our study between HCC and cognitive decline. Recent studies investigating the functions of the hippocampus along its longitudinal axis concluded that the tail is most sensitive to ischemia and that the dentate gyrus is the most likely place where age related cognitive decline could start.[8–10] Possibly the calcifications as seen on CT are related to VFC and reflect these aging related changes. VFC was also found in patients with Alzheimer’s disease. However, Alzheimer’s disease is thought to originate in the enthorhinal cortex which is located around the head of the hippocampus. Thus, based on the location in the hippocampus, we believe that the calcifications as visualized in our study, mainly localized in the tail, are more related to aging related cognitive decline. However, more in-depth studies are required to ensure that the calcification patterns in cognitive decline are distinct from the calcification pattern in Alzheimer’s disease.

CT and MRI are currently the only radiological modalities that are capable of visualizing calcifications in the brain. MRI is the most frequently utilized imaging modality in neurocognitive studies because of the high spatial resolution, soft tissue contrast, functional imaging capabilities and lack of ionizing radiation. The literature on imaging of intracranial calcifications using MRI is sparse because of inherent limitations of the modality.[3] Calcifications on MRI have various signal intensities on conventional T_1_- and T_2_-weighted acquisitions which makes definitive identification of calcifications difficult.[11,12] The best MRI acquisition technique for the detection of calcification would be susceptibility weighted imaging (SWI), however, in our center, SWI is not part of a standard ‘cognitive imaging package’. Also, signal intensities of calcification overlap with those of other signal sources (e.g. blood and air). The accuracy of SWI in the detection of hippocampal calcification has not been assessed so far.

CT scans are used far less frequently in neurocognitive research. CT is based on x-ray attenuation of tissues and excels in visualizing high contrast areas. Because there is little variation in the density of brain tissue CT scans cannot pick up on subtle differences which MRI can. On the other hand, dense and high contrast tissues like calcifications are easily distinguished. While MRI will remain the primary modality for neurocognitive studies, with the increasing evidence on prognostic CT markers for cognitive decline and stroke and the dramatic decrease in radiation dose, CT could see an increase in applicability in this field.[13–15]

The presented study is limited by the fact that the cognitive status of the majority of the control group was unknown. History of dementia was reported in four patients of the control group. These patients did not show HCC and our reported OR could therefore be slightly overestimated. Secondly the sample size of the study was relatively small which precluded multivariable analysis. However, the difference in presence of HCC between cases and control was substantial which supports the validity of our results.

In conclusion, we found a significantly higher prevalence of HCC on CT in patients referred to a memory clinic for cognitive problems compared to matched controls and HCC was associated with worse cognitive functioning. Larger studies will be required to further analyze and determine the role of HCC in cognitive decline and its relation to vascular disease.
